# Primary tumor sidedness is an independent prognostic marker for survival in metastatic colorectal cancer: Results from a large retrospective cohort with mutational analysis

**DOI:** 10.1002/cam4.1558

**Published:** 2018-05-17

**Authors:** Sophia C. Kamran, Jeffrey W. Clark, Hui Zheng, Darrell R. Borger, Lawrence S. Blaszkowsky, Jill N. Allen, Eunice L. Kwak, Jennifer Y. Wo, Aparna R. Parikh, Ryan D. Nipp, Janet E. Murphy, Lipika Goyal, Andrew X. Zhu, A. John Iafrate, Ryan B. Corcoran, David P. Ryan, Theodore S. Hong

**Affiliations:** ^1^ Harvard Radiation Oncology Program Boston MA USA; ^2^ Division of Hematology and Oncology Massachusetts General Hospital Boston MA USA; ^3^ Biostatistics Massachusetts General Hospital Boston MA USA; ^4^ Department of Radiation Oncology Massachusetts General Hospital Boston MA USA; ^5^ Department of Pathology Massachusetts General Hospital Boston MA USA

**Keywords:** colorectal cancer, EGFR inhibitors, mutational profiling, overall survival, primary tumor sidedness

## Abstract

Recent reports demonstrate inferior outcomes associated with primary right‐sided vs left‐sided colorectal tumors in patients with metastatic colorectal cancer (mCRC). We sought to describe our experience with mCRC patients on whom we have molecular data to determine whether primary tumor sidedness was an independent prognostic marker for overall survival (OS). mCRC patients with documented primary tumor sidedness who received mutational profiling between 2009 and 2014 were identified (n = 367, median follow‐up 30.4 months). Mutational profiling for >150 mutations across commonly mutated cancer genes including *RAS, PIK3CA, BRAF,* and *PTEN* as well as treatment data, including receipt of a biologic agent, were collected. Univariable/multivariable models were used to analyze relationships between collected data and OS. Among 367 patients, sidedness breakdown was as follows: 234 left (64%), 133 right (36%). 56% were male, with a median age at diagnosis of 57 (range 24‐89). A total of 143 patients had *RAS* mutations. Five‐year OS was 41%, median OS was 54 months (range 1‐149). Five‐year OS for left‐ vs right‐sided tumors was 46% vs 24% (*P* < .0001). On univariable analysis, among both *RAS* wildtype and mutant tumors, left‐sided tumors continued to have improved OS vs right‐sided tumors (HR: 0.49, 95% CI: 0.34‐0.69 *RAS* wildtype; HR: 0.61, 95% CI: 0.40‐0.95 *RAS* mutant). Left‐sidedness was an important prognostic factor for OS among *RAS* wildtype patients despite treatment with or without a biologic agent (*P* < .05). Left‐sidedness remained significant for improved OS on multivariable analysis (*P* < .0001). Left‐sided primary tumor remained most important prognostic factor for OS, even when adjusting for mutational status and receipt of biologic agent.

## 1 INTRODUCTION

Colorectal cancer is a heterogenous disease, with an estimated 140 250 new cases and 50 630 deaths in 2018.^1^ Currently, it is the second leading cause of death among American men and third leading cause of death among American women. Despite decreased trends noted among screened individuals 50 years and older, rates of colorectal cancer have increased approximately 2% per year between 1993 and 2013 in individuals younger than age 50 making it an increasingly important disease problem in younger patients.^1^

Metastatic colorectal cancer is a similarly heterogeneous disease, with a poor long‐term survival rate (5‐year survival rate of <15%).^2^, ^3^ However, advancements in chemotherapeutics and other treatment strategies has improved median survival from 12 months to up to 30 months.^4^ The molecular pathways underlying the development of colorectal cancer have been extensively studied;^5^, ^6^, ^7^ however, there have been few identified prognostic biomarkers for outcomes. Current prognostic biomarkers include germline mutations in DNA mismatch repair genes in stage II/III disease, and *BRAF* ^*V600E*^ mutations in stage IV disease.^8^, ^9^, ^10^ Recently, single institutional analyses have demonstrated a significant difference in prognosis between patients who present with right‐sided primary tumors vs left‐sided primary tumors, with the latter having improved outcomes.^11^, ^12^, ^13^ There have also been reports of primary tumor sidedness playing an important prognostic and predictive role in metastatic colorectal cancer patients treated with biological agents in clinical trials with improved outcomes for left‐sided tumors.^13^, ^14^, ^15^, ^16^, ^17^, ^18^

The purpose of our study was to evaluate our institutional experience with metastatic colorectal cancer patients who also underwent clinical mutational profiling for evaluation of prognostic and predictive factors of outcomes. We also evaluated the predictive role of primary tumor sidedness on biologic agent effectiveness.

## 2 METHODS

### 2.1 Patient selection

Under an institutional review board‐approved protocol, 717 patients with a diagnosis of metastatic colorectal cancer who received mutational profiling as part of their standard of care between 2009 and 2014 were identified. Medical records were reviewed for patient, tumor, and treatment characteristics and clinical outcomes. Patients had initial diagnoses between 1980 and 2014 and were diagnosed with metastatic cancer between 2001 and 2014. Patients were part of an original study looking at body mass index (BMI),^19^, ^20^ and excluded if there was no total sidedness documented for a total of 392 patients identified. Distinction between colon and rectal cancer was based on location of tumor proximal or distal to the rectosigmoid junction. Further tumor location breakdown by side was as follows: left, splenic flexure to rectum; right, cecum to hepatic flexure; and transverse, hepatic to splenic flexure. Patients with transverse primary tumors were removed, leaving a final study population of 367 patients.

### 2.2 Mutational analysis

Tumor genotyping was performed on all 367 patients. Nucleic acids were extracted from diagnostic formalin‐fixed, paraffin‐embedded tumor tissue using a modified FormaPure system (Agencourt Bioscience Corporation, Beverly, MA) on a custom Beckman Coulter Biomek NX^P^ workstation (Beckman Coulter, Pasadena, CA). Mutational profiling queried over >150 commonly mutated loci across 23 cancer genes, including v‐akt murine thymoma viral oncogene homolog 1 (*AKT1*); adenomatosis polyposis coli (*APC*); *BRAF*; catenin (cadherin‐associated protein) β1, 77 kDa (*CTNNB1*); *EGFR*; v‐erb‐b2 avian erythroblastic leukemia viral oncogene homolog 2 (*ERBB2*); isocitrate dehydrogenase 1 (NAPD+), soluble (*IDH1*); v‐kit Hardy‐Zuckerman 4 feline sarcoma viral oncogene homolog (*KIT*); rat sarcoma viral oncogene homolog (*RAS*); mitogen‐activated protein kinase kinase 1 (*MAP2K1*); notch 1 (*NOTCH1*); neuroblastoma *RAS* viral oncogene homolog (*NRAS*); phosphatidylinositol‐4,5‐bisphosphate 3‐kinase, catalytic subunit α (*PIK3CA*); phosphatase and tensin homolog (*PTEN*); and tumor protein 53 (*TP53*).^21^ This was performed using a custom‐modified ABI PRISM SNaPshot Multiplex System (Applied Biosystems/Life Technologies Corporation, Carlsbad, Calif), as previously described.^22^ Testing of tumor suppressor genes *TP53, APC,* and *PTEN* was limited to the most common mutation sites (limiting coverage to 29%, 15%, and 15% respectively, of all known somatic mutations).^22^ For testing performed after 5/8/2014, the assay was converted to next‐generation sequencing.

### 2.3 Statistical analysis

All statistical analyses were performed using R version 3.3.2 or SAS 9.4. Univariable Cox regression analyses and multivariable Cox regression analyses were built using stepwise variable selection modeling in SAS (criteria *P* = .05). Clinically relevant variables (Table 1) were used to identify clinical or molecular features associated with the outcome of overall survival (OS). OS was calculated from date of initial diagnosis to date of death. The Kaplan‐Meier method was used to generate actuarial survival estimates for OS. *P*‐values were considered significant for <.05.

**Table 1:** Demographic information

Characteristic	N = 367 (%)
Gender	
Female	163 (44%)
Male	204 (56%)
Age at diagnosis (median, range)	57.0 (24‐89)
Sidedness of colorectal cancer	
Left	234 (64%)
Right	133 (36%)
Further left‐sided breakdown	[N = 234 (%)]
Splenic flexure to descending colon	21 (9%)
Sigmoid	75 (32%)
Rectum	138 (59%)
Smoking status	
Former	145 (40%)
Current	33 (9%)
Never	189 (51%)
Diabetes at diagnosis	
No	312 (85%)
Yes	55 (15%)
BMI at diagnosis (median, range)	26.8 (16.8‐72)
Stage 4 at presentation	258 (70%)
One metastatic organ	231 (63%)
>1 metastatic organ	136 (37%)
Metastatic sites	
Liver	276
Lung	110
Abdominal lymph nodes	73
Bone	27
Brain	9
Other	22
Surgery for primary disease	
Yes	244 (67%)
No	114 (31%)
Unknown	9 (2%)
Chemotherapy received	
Yes	344 (94%)
No	16 (41%)
Unknown	7 (2%)
Definitive radiation to primary	
Yes	79 (22%)
No	282 (76%)
Unknown	6 (2%)
FOLFOX received	
Yes	286 (78%)
No	74 (20%)
Unknown	7 (2%)
FOLFIRI received	
Yes	170 (46%)
No	190 (52%)
Unknown	7 (2%)
EGFR inhibitor received	
Yes	100 (27%)
No	260 (71%)
Unknown	7 (2%)
Bevacizumab received	
Yes	173 (47%)
No	187 (51%)
Unknown	7 (2%)
Microsatellite Instability (MSI) status	
MSI high (MSI‐H)	21 (6%)
Stable (MSS)	173 (47%)
Unknown	173 (47%)
Mutation present	
Yes	258 (70%)
No	109 (30%)

## 3 RESULTS

### 3.1 Patient demographic and treatment characteristics

**Figure 1:**
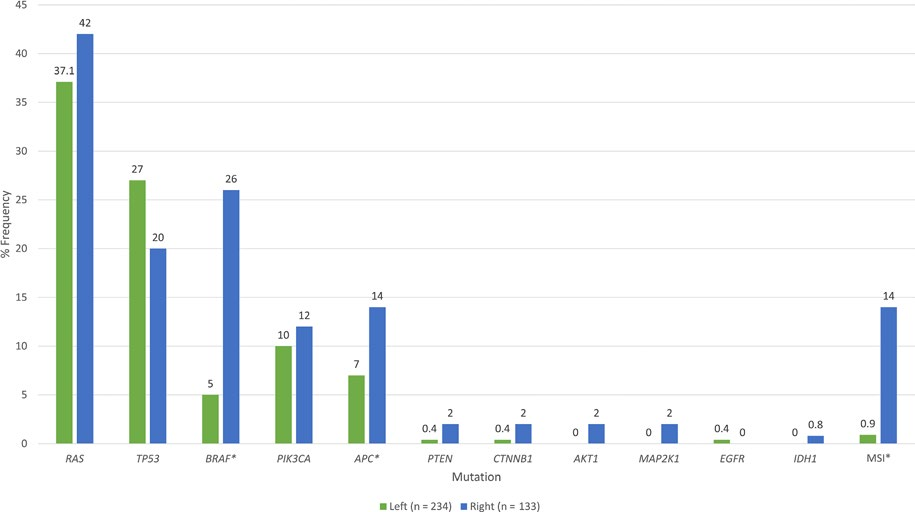
Mutation frequency among left vs right‐sided tumors. Right‐sided tumors were more likely to harbor *BRAF* and *APC* mutations, as well as demonstrate microsatellite instability (MSI). *Statistically significant (p<0.05)

Of the 367 patients identified, sidedness breakdown was as follows: 234 left (64%), 133 right (36%) (Table 1). Further breakdown of those patients with left‐sided tumors (n = 234) was as follows: splenic flexure to descending colon, 21 (9%); sigmoid, 75 (32%); and rectum, 138 (59%). Of the entire cohort, median age at diagnosis was 57 years (range, 24‐89), 204 of the patients were male (56%), and 178 patients (49%) were either former or current smokers at time of diagnosis. Most did not have diabetes at the time of diagnosis (85%), and median BMI was 26.8 (range, 16.8‐72). Seventy percent were stage 4 at initial diagnosis. For mutational testing, 143 patients (39%) were *RAS* mutant, and 47 patients (13%) had *BRAF* mutations (Table S1). Mutational breakdown between left‐sided vs right‐sided tumors is depicted in Figure 1. *BRAF* and *APC* mutations were significantly associated with right‐sided tumors (*P* < .05).

Sixty‐seven percent of patients underwent surgery for their primary tumor in the colon. Of those, 145 patients (59%) received surgery for their primary tumor with known metastatic disease, while 99 patients (41%) received surgery for their primary tumor before development of metastases. Most received chemotherapy (94%), and 79 patients (22%) received definitive radiation to the primary tumor in the colon. Of those that received definitive radiation, 41 patients (52%) received radiation for their primary tumor with known metastatic disease elsewhere (of which 31/41 patients had primary rectal tumors), while 38 patients (48%) received radiation for their primary tumor before development of metastases (of which 30/38 patients had primary rectal tumors). For chemotherapy, 78% received leucovorin, fluorouracil, and oxaliplatin (FOLFOX), 46% received leucovorin, fluorouracil, and irinotecan (FOLFIRI). One hundred patients (27%) received an EGFR inhibitor, while 173 patients (47%) received bevacizumab, in addition to their chemotherapy.

### 3.2 Outcomes

Median follow‐up was 30.4 months (range, 1‐149) for the entire cohort. Five‐year OS was 41%, and median OS was 54.1 months (range, 1‐149).

### 3.3 Predictors of outcomes: Univariable and multivariable analyses

#### 3.3.1 Overall survival

**Figure 2:**
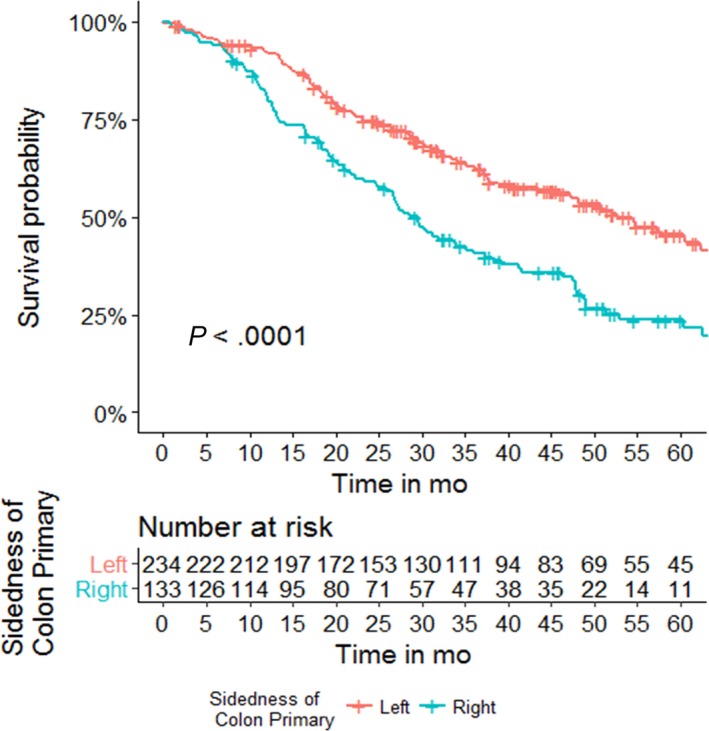
Overall survival by primary tumor sidedness. Left‐sided tumors had improved overall survival compared to right‐sided tumors (HR: 0.54, 95% CI: 0.41‐0.70, *P* < .0001). Three‐year OS 46% vs 24% left‐sided vs right‐sided tumors, respectively (*P* < .0001)

Stage 4 disease at time of diagnosis (HR: 1.92 95% CI: 1.42‐2.61, *P* < .0001), microsatellite instability (MSI) high status (HR: 1.83, 95% CI: 1.07‐3.15, *P* = .03), or harboring a *BRAF* (HR: 1.95, 95% CI: 1.35‐2.28, *P* = .0004) are all associated with a worse OS on univariable analysis (Table 2). Left‐sided tumors (HR: 0.54, 95% CI: 0.41‐0.70, *P* < .0001) (Figure 2), surgical resection of the primary disease (HR: 0.46, 95% CI: 0.34‐0.62, *P* < .0001), and definitive radiation to the primary disease (HR: 0.51, 95% CI: 0.36‐0.74, *P* = .0003) are associated with improved OS. When controlling for those who presented with metastatic disease, both surgical resection (HR: 0.53, 95% CI: 0.38‐0.74, *P* = .00014) and definitive radiation (HR: 0.37, 95% CI: 0.21‐0.64, *P* = .00042) of the primary disease continued to confer a survival benefit. On multivariable, adjusted analysis, left‐sided tumors (HR: 0.48, 95% CI: 0.36‐0.65, *P* < .0001), and primary surgical resection (HR: 0.37, 95% CI: 0.28‐0.50, *P* < .0001) remained significant for improved OS, while harboring a *BRAF* mutation remained significant for worse OS (HR: 1.68, 95% CI: 1.13‐2.50, *P* = .01) (Table 3).

**Table 2:** Univariable analysis of predictors for overall survival (OS)

N = 367, 217 (59%) deaths	OS univariate hazard ratio (95% CI)	*P*‐value
Gender		
Female vs male	1.06 (0.81, 1.38)	.67
Age at diagnosis	1.02 (1.00, 1.03)	.01
Left‐sided tumors vs right‐sided tumors	0.54 (0.41, 0.70)	**<.0001**
Smoking status^a^	1.04 (0.80, 1.36)	.76
Diabetes at diagnosis	0.98 (0.67, 1.44)	.91
BMI at diagnosis	0.98 (0.96, 1.01)	.15
Stage 4 at presentation	1.92 (1.42, 2.61)	**<.0001**
Surgery for primary disease	0.46 (0.34, 0.62)	**<.0001**
Chemotherapy received	0.69 (0.35, 1.33)	.28
Definitive radiation to primary	0.51 (0.36, 0.74)	**.0003**
FOLFOX received	0.76 (0.55, 1.03)	.09
FOLFIRI received	1.09 (0.84, 1.43)	.52
EGFR inhibitor received	1.11 (0.83, 1.47)	.48
Bevacizumab received	0.97 (0.74, 1.27)	.81
MSI status (high vs stable)	1.83 (1.07, 3.15)	**.03**
*RAS*	1.17 (0.88, 1.54)	.28
*TP53*	0.81 (0.59, 1.12)	.20
*BRAF*	1.95 (1.35, 2.82)	**.0004**
*PIK3CA*	0.83 (0.52, 1.33)	.44
*APC*	1.03 (0.63, 1.66)	.92
*PTEN*	2.00 (0.64, 6.26)	.23
*CTNNB1* b	‐	.98
*AKT1*	9.41 (2.29, 38.75)	**.0019**
*MAP2K1* b	‐	.98
*EGFR* b	‐	.98
*IDH1*	1.19 (0.17, 8.54)	.85

a Current/former vs never.

b Too few events for convergence.

Bold values refer to p‐value statistical significance.

**Table 3:** Multivariable Cox analysis of predictors for overall survival (OS) for metastatic colorectal cancer patients, n = 367

	HR (95% CI)	*P*‐value
Left‐sided tumors vs right‐sided tumors	0.48 (0.36, 0.65)	**<.0001**
Surgery for primary disease	0.37 (0.28, 0.50)	**<.0001**
*BRAF*	1.68 (1.13, 2.50)	**.01**

Bold values refer to p‐value statistical significance.

#### 3.3.2 Subgroup analyses—overall survival

On subgroup analysis for overall survival, left‐sided *RAS* wildtype patients had improved OS, compared to right‐sided *RAS* wildtype patients (HR: 0.49, 95% CI: 0.34‐0.69, *P* < .0001) (Table 4, Figure S1A). Left‐sided *RAS* mutation patients also had improved OS, compared to right‐sided *RAS* mutation patients (HR: 0.61, 95% CI: 0.40‐0.95, *P* = .03) (Figure S1B). Patients with left‐sided, *RAS* wildtype, *BRAF* wildtype tumors had improved OS, compared to patients with right‐sided, *RAS* wildtype, *BRAF* wildtype tumors (HR: 0.54, 95% CI: 0.35‐0.83, *P* = .005) (Figure S2A), but there was no significant difference in survival between left‐ and right‐sided tumors among patients with *RAS* wildtype, *BRAF* mutant tumors (Figure S2B). Among those who had left‐sided tumors and received an EGFR inhibitor or bevacizumab, vs those with left‐sided tumors that did not receive an EGFR inhibitor or bevacizumab, there was no statistically significant difference in OS (Figure S3). This was similar among patients with right‐sided tumors (Figure S4). Further stratifying on *RAS* mutation status, there was no significant difference in survival among patients with left‐sided, *RAS* wildtype tumors who received an EGFR inhibitor or not (*P* = .32) or bevacizumab or not (*P* = .94) (Figure S5). Among patients with right‐sided, *RAS* wildtype tumors, there was a trend toward worse OS among those who received an EGFR inhibitor vs those who did not (HR: 1.63, 95% CI: 0.95‐2.81, *P* = .08) (Figure S6A). There was no statistically significant difference in this group among those who received bevacizumab vs not (Figure S6B). Among those patients with *RAS* wildtype tumors, left‐sidedness was an important prognostic factor among those who did or did not receive an EGFR inhibitor (vs right‐sided tumors, HR: 0.36, 95% CI: 0.21‐0.62, *P* < .0001 and HR: 0.51, 95% CI: 0.31‐0.82, *P* = .005, respectively) (Figure S7). Similarly, there was a statistically significant trend toward improved OS among patients with *RAS* wildtype tumors who received bevacizumab, comparing left side vs right side (HR: 0.47, 95% CI: 0.29‐0.73, *P* = .001) and among those who did not receive bevacizumab, comparing left side vs right side (HR: 0.52, 95% CI: 0.30‐0.90, *P* = .02) (Figure S8). On analysis of all left‐side vs right‐side tumors in patients who received bevacizumab, regardless of *RAS* status, left‐sided tumors had improved OS (HR: 0.65, 95% CI: 0.44‐0.93, *P* = .02) (Figure S9).

**Table 4:** Subgroup analyses for overall survival (OS)

Subgroup analysis	OS univariate hazard ratio (95% CI)	*P*‐value
N = 224, 134 (60%) deaths		
Left‐sided *RAS* WT tumors (vs right‐sided *RAS* WT tumors)	0.49 (0.34, 0.69)	**<.0001**
N = 143, 83 (58%) deaths		
Left‐sided *RAS* mutant tumors (vs right‐sided *RAS* mutant tumors)	0.61 (0.40, 0.95)	**.03**
N = 177, 100 (56%) deaths		
Left‐sided *RAS* WT, *BRAF* WT tumors (vs right‐sided *RAS* WT, *BRAF* WT tumors)	0.54 (0.35, 0.83)	**.005**
N = 47, 34 (72%) deaths		
Left‐sided *RAS* WT, *BRAF* mutant tumors (vs right‐sided *RAS* WT, *BRAF* mutant tumors)	0.73 (0.32, 1.68)	.45
N = 159, 82 (52%) deaths		
Rectal tumors vs left‐sided tumors	1.25 (0.66, 2.37)	.50
N = 213, 110 (52%) deaths		
Rectal tumors vs sigmoid tumors	1.42 (0.96, 2.12)	.08
N = 234, 121 (52%) deaths		
Left‐sided EGFR inhibitor received (vs left‐sided no EGFR inhibitor received)	1.20 (0.83, 1.73)	.34
Left‐sided bevacizumab received (vs left‐sided no bevacizumab received)	1.19 (0.82, 1.72)	.35
N = 133, 96 (72%) deaths		
Right‐sided EGFR inhibitor received (vs right‐sided no EGFR inhibitor received)	1.35 (0.85, 2.14)	.21
Right‐sided bevacizumab received (vs right‐sided no bevacizumab received)	0.73 (0.44, 1.20)	.13
N = 143, 75 (52%) deaths		
Left‐sided *RAS* WT EGFR inhibitor received (vs left‐sided *RAS* WT no EGFR inhibitor received)	1.27 (0.80, 2.02)	.32
Left‐sided *RAS* WT bevacizumab received (vs left‐sided *RAS* WT no bevacizumab received)	1.02 (0.64, 1.62)	.94
N = 77, 58 (75%) deaths		
Right‐sided *RAS* WT EGFR inhibitor received (vs right‐sided *RAS* WT no EGFR inhibitor received)	1.63 (0.95, 2.81)	.08
Right‐sided *RAS* WT bevacizumab received (vs right‐sided *RAS* WT no bevacizumab received)	1.05 (0.62, 1.78)	.86
N = 90, 65 (72%) deaths		
Left‐sided *RAS* WT EGFR inhibitor received (vs right‐sided *RAS* WT EGFR inhibitor received)	0.36 (0.21, 0.62)	**<.0001**
N = 130, 68 (52%) deaths		
Left‐sided *RAS* WT no EGFR inhibitor received (vs right‐sided *RAS* WT no EGFR inhibitor received)	0.51 (0.31, 0.82)	**.005**
N = 110, 77 (70%) deaths		
Left‐sided *RAS* WT bevacizumab received (vs right‐sided *RAS* WT bevacizumab received)	0.47 (0.29, 0.73)	**.001**
N = 110, 56 (51%) deaths		
Left‐sided *RAS* WT no bevacizumab received (vs right‐sided *RAS* WT no bevacizumab received)	0.52 (0.30, 0.90)	**.02**

WT=wildtype.

Bold values refer to p‐value statistical significance.

## 4 DISCUSSION

In this study, we evaluated the prognostic utility of sidedness in metastatic colorectal cancer patients with mutational profiling data. We found that primary tumor sidedness was strongly associated with overall survival on univariable and multivariable analysis, when accounting for both clinical and treatment factors, including mutational status. Primary tumor sidedness also was more important for OS outcome despite receipt of a biologic agent, when accounting for mutational status.

Metastatic colorectal cancer is a heterogenous disease, with heterogenous responses to treatment. Other than the presence of *RAS* mutations and non‐responsiveness to anti‐EGFR monoclonal antibody therapy, biomarkers for treatment response and outcomes are lacking, with few identified. Recent reports have suggested that primary tumor sidedness plays a significant role in prognostication for metastatic colon cancer, with left‐sided tumors having improved outcomes.^11^, ^12^, ^13^, ^14^, ^15^, ^16^, ^17^, ^18^ The two sites differ biologically as well, perhaps related to their difference in embryological origin. Right‐sided tumors are more frequently diploid, characterized by mucinous histology, more frequently harbor high microsatellite instability, CpG island methylation, and *BRAF* mutations.^10^, ^23^, ^24^, ^25^, ^26^ Left‐sided tumors are infiltrative, and most often have chromosomal instability.^23^, ^24^, ^25^

Notably, an analysis of a first‐line study comparing chemotherapy plus cetuximab vs chemotherapy plus bevacizumab reported improved results for cetuximab in patients with left‐sided tumors, while patients with right‐sided tumors benefit from the addition of bevacizumab.^14^ Separately, an analysis of the NCIC CTGCO.17 trial found that adding cetuximab to best supportive care in patients with chemotherapy‐refractory *KRAS* wildtype disease significantly benefitted patients with left‐sided tumors, with limited benefit to patients with right‐sided disease.^15^ In an analysis of the CRYSTAL and FIRE‐3 studies with patients with *RAS* wildtype tumors, the addition of cetuximab only significantly benefitted patients with left‐sided tumors.^16^ Our results found that, when comparing left‐sided *RAS* wildtype tumors to right‐sided *RAS* wildtype tumors, there was a survival benefit associated among patients with left‐sided tumors who received EGFR inhibitor therapy (HR: 0.36, *P* < .0001). Among those who did not receive EGFR inhibitor therapy, left‐sidedness continued to be significantly associated with an improved OS benefit (HR: 0.51, *P* = .005). There were no statistically significant findings when comparing patients with left‐sided tumors who received an EGFR inhibitor or not, nor when comparing patients with left‐sided *RAS* wildtype tumors who received an EGFR inhibitor or not. When comparing patients with right‐sided tumors with regard to receipt of an EGFR inhibitor, there was no significant difference in outcome. However, among patients with right‐sided *RAS* wildtype tumors, there was a trend toward worse survival among those who received an EGFR inhibitor (HR: 1.63, *P* = .08). Our results are consistent with previous studies,^14^, ^15^, ^16^ and previous research has demonstrated that an EGFR inhibitor‐sensitive phenotype is more prevalent in left‐sided tumors, with a subset of right‐sided tumors harboring this sensitivity.^27^ However, our study found that despite receipt of an EGFR inhibitor, even if harboring *RAS* wildtype mutational status, sidedness plays the most important role in overall survival.

With regards to anti‐angiogenic therapy, the data are mixed. One study reported improved outcomes among those with left‐sided tumors originating from the sigmoid colon or rectum,^17^ while another failed to find a significant association.^18^ He et al^13^ found that only left‐sided tumors benefited from anti‐angiogenic therapy such as bevacizumab. When comparing left‐ vs right‐sided primary tumor location in patients with *RAS* wildtype tumors, left‐sided tumors were significantly associated with a survival benefit (HR: 0.47, *P* = .001) among those who received bevacizumab. Similarly, left‐sided tumors were significantly associated with a survival benefit among those who did not receive any bevacizumab (HR: 0.52, *P* = .02). When comparing left‐ vs right‐sided primary tumor location in all patients who received bevacizumab regardless of *RAS* status, left‐sided tumors continued to be significantly associated with a survival benefit (HR: 0.65, *P* = .02).

In addition, our findings demonstrate a survival benefit among those who underwent surgical resection for their primary tumor. This remained significant when controlling for patients who presented with metastatic disease and underwent primary tumor resection. Data have been mixed on the utility of primary resection for metastatic colorectal cancer. Primary tumor resection is largely reserved for those experiencing obstruction or severe bleeding, as a palliative tool.^28^ A Cochrane collaboration in 2012 analyzed seven non‐randomized clinical studies, for a total of 1086 patients. Of these patients, 722 were treated with surgical resection for their primary tumor, while 364 did not receive surgical resection. There was no difference in survival between the two groups.^29^ However, other studies have demonstrated a survival benefit in primary tumor resection among patients with metastatic colorectal cancer.^30^, ^31^, ^32^ Current randomized studies are investigating the utility of upfront primary surgical resection (CAIRO4,^33^ SYNCHRONOUS,^34^ Korean NCT01978249^35^), the results of which are greatly anticipated to further elucidate this question.

This study must be interpreted in the context of several limitations. The study is retrospective in nature and therefore subject to inherent biases. The sample size is small, resulting in insufficient power, thus requiring further validation in a larger, external cohort. In addition, the mutational profiling is limited and does not capture all known mutations in colorectal cancer. However, despite these limitations, the results are compelling in that they demonstrate that tumor sidedness has the most significant correlation with outcomes, including patients who receive different chemotherapy treatments and biologic agents such as EGFR inhibitors or anti‐angiogenesis therapeutics. Furthermore, this association is found off a prospective trial in clinical scenarios which may be more consistent with real‐world practice.

## 5 CONCLUSION

Our findings further confirm that primary tumor sidedness plays an important role in prognosticating outcome among patients with metastatic colorectal cancer and remained significant when adjusting for *RAS* mutation status as well as receipt of biologic agents. Future trials should incorporate primary tumor sidedness in patient stratification, and research efforts should focus on harnessing the differences in biology between the two sites to improve personalized medicine for patients with metastatic colorectal cancer.

## CONFLICT OF INTEREST

All authors declare no conflicts of interest.
