# Supplementary material for: Primary tumor sidedness is an independent prognostic marker for survival in metastatic colorectal cancer: Results from a large retrospective cohort with mutational analysis
Source: Cancer Med. 2018 May 17;7(7):2934–42. doi: 10.1002/cam4.1558 (PMC6051212; doi:10.1002/cam4.1558)
Supplement: Supplementary file 1 [file CAM4-7-2934-s001.docx]

**Supplemental Materials**

**TABLES**

**Table 1S. Mutational profiling**

| **Mutation** | **N =367 (%)** |
| --- | --- |
| *RAS* | 143 (39%) |
| *TP53* | 90 (25%) |
| *BRAF* | 47 (13%) |
| *PIK3CA* | 39 (11%) |
| *APC* | 34 (9%) |
| *PTEN* | 3 (1%) |
| *CTNNB1* | 3 (1%) |
| *AKT1* | 2 (1%) |
| *MAP2K1* | 2 (1%) |
| *EGFR* | 1 (1%) |
| *IDH1* | 1 (1%) |

**FIGURES**

**Supplemental Figure 1S.**

**
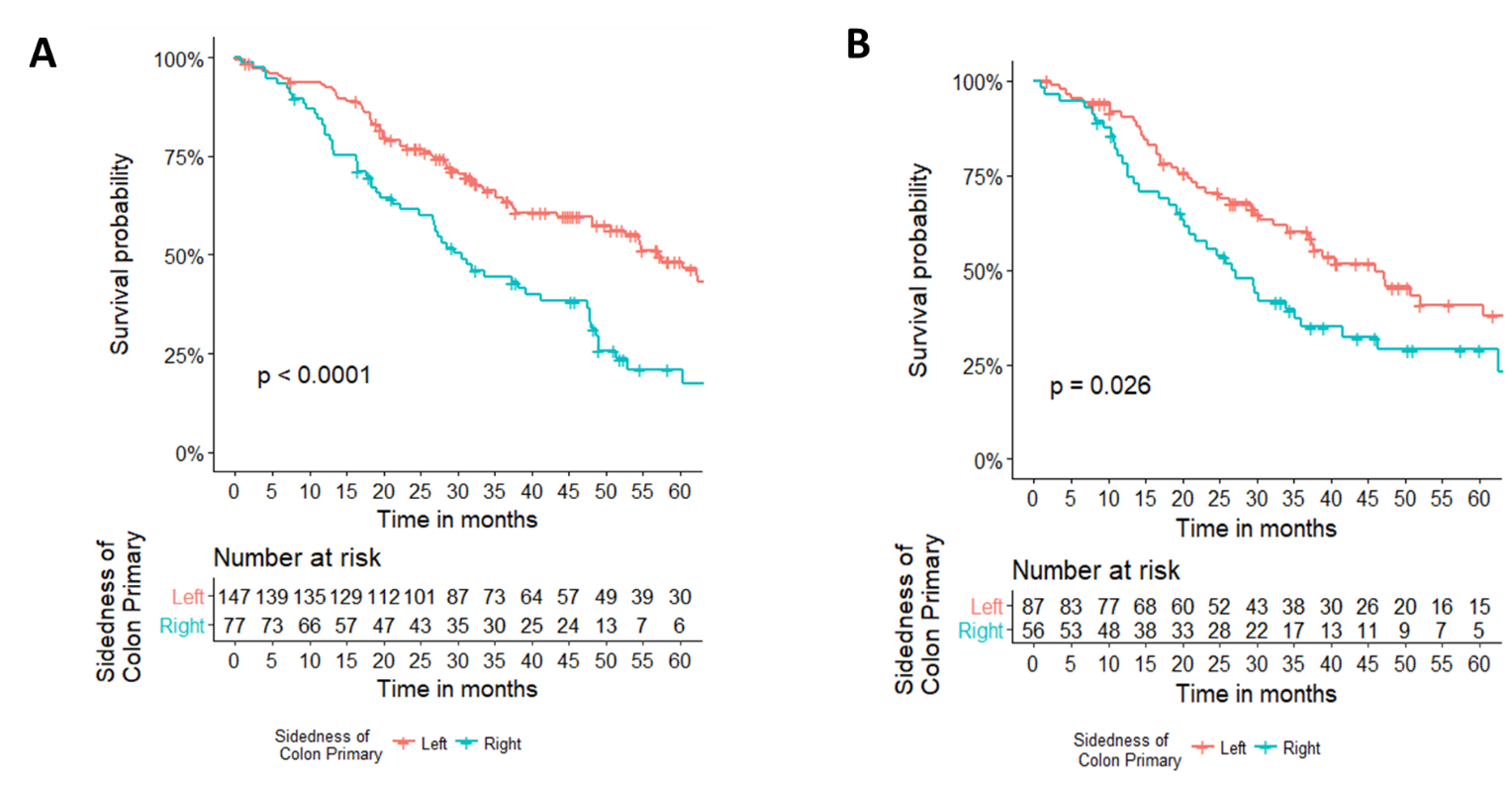
**

**A. Overall survival by sidedness among *RAS* WT tumors.** Left-sided *RAS* wildtype patients had improved OS, compared to right-sided *RAS* wildtype patients (HR 0.49, 95%CI 0.34-0.69, p<0.0001). Five-year OS 48% versus 21%, left-sided versus right-sided tumors respectively.

**B. Overall survival by sidedness among *RAS* mutant tumors.** Left-sided *RAS* mutation patients had improved OS, compared to right-sided *RAS* mutation patients (HR 0.61, 95%CI 0.40-0.95, p=0.03). Five-year OS 41% versus 29%, left-sided versus right-sided tumors respectively.

**Supplemental Figure 2S.**

**
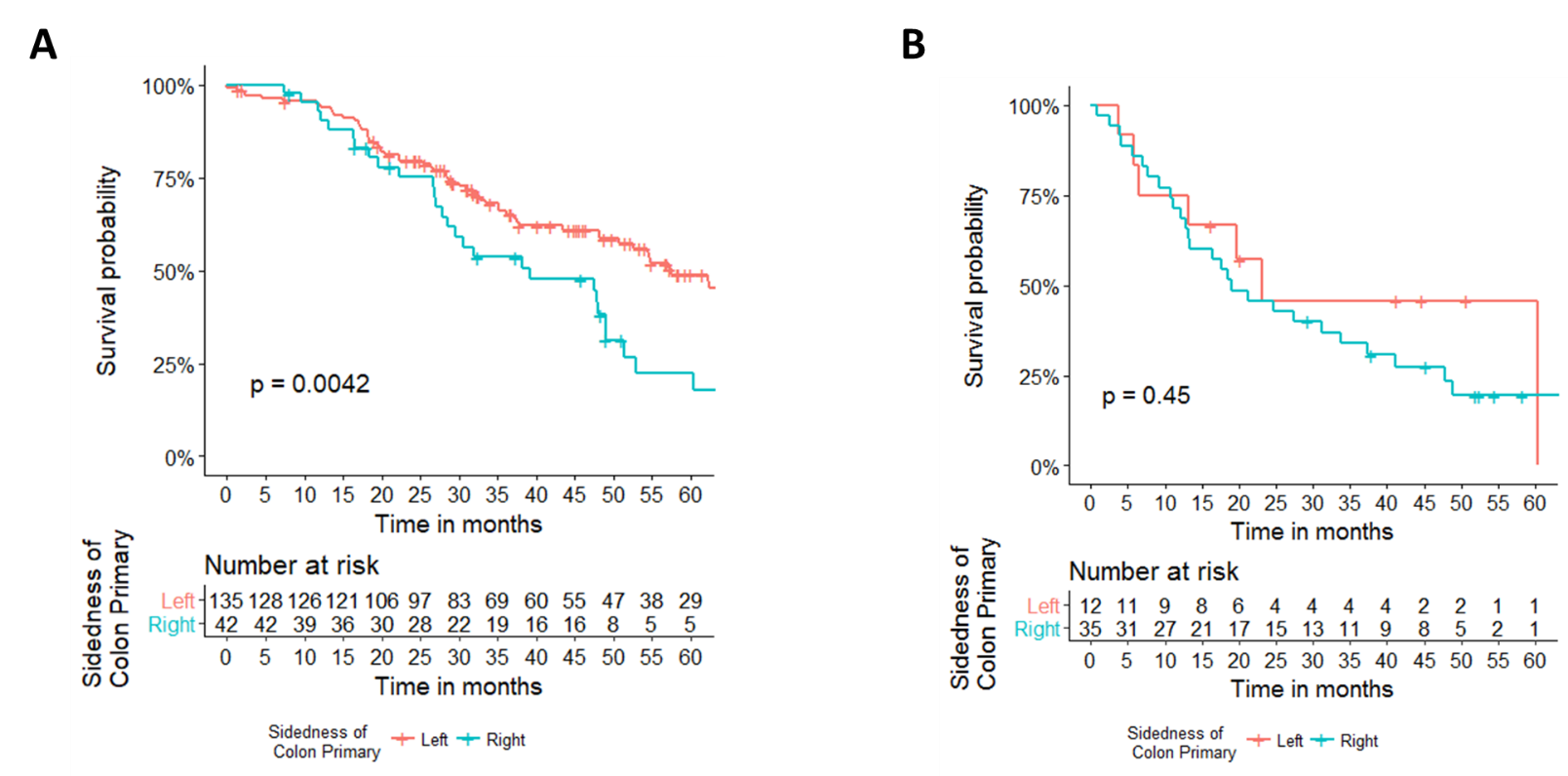
**

**A. Overall survival by sidedness among *RAS* WT, *BRAF* WT tumors.** Patients with left-sided, *RAS* wildtype, *BRAF* wildtype tumors had improved OS, compared to patients with right-sided, *RAS* wildtype, *BRAF* wildtype tumors (HR 0.54, 95%CI 0.35-0.83, p=0.005). Five-year OS 49% versus 22% left-sided versus right-sided tumors, respectively.

**B. Overall survival by sidedness among *RAS* WT, *BRAF* mutant tumors.** There was no significant difference in survival between left and right-sided tumors among patients with *RAS* wildtype, *BRAF* mutant tumors (p=NS). Five-year OS 46% versus 20% left-sided versus right-sided tumors, respectively.

**Supplemental Figure 3S.**

**
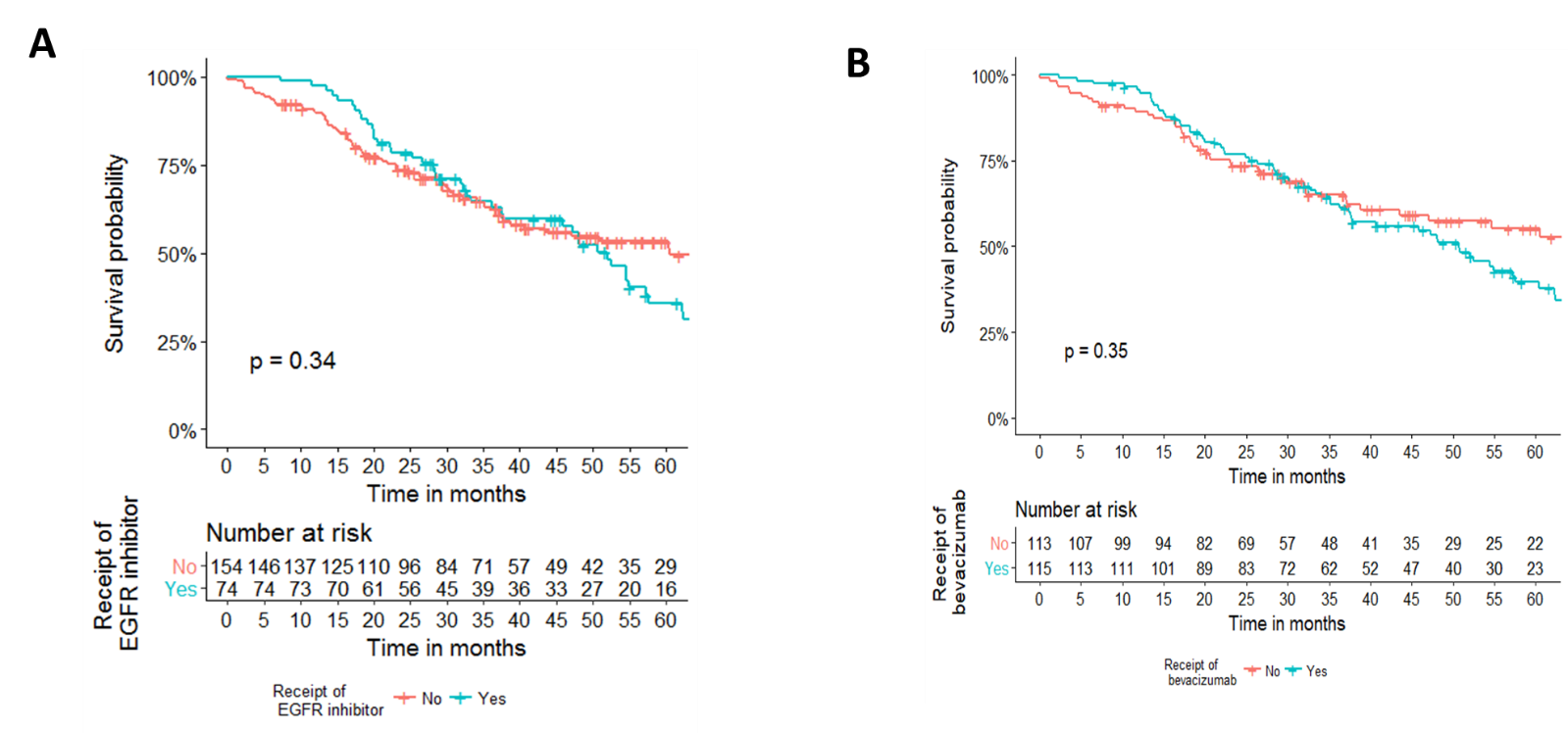
**

**A. Impact of receipt of EGFR inhibitor on OS among left-sided tumors.** Five-year OS 53% versus 36% no receipt versus receipt, respectively, p=NS.

**B. Impact of receipt of bevacizumab on OS among left-sided tumors.** Five-year OS 55% versus 39% no receipt versus receipt, respectively, p=NS.

**Supplemental Figure 4S.**

**
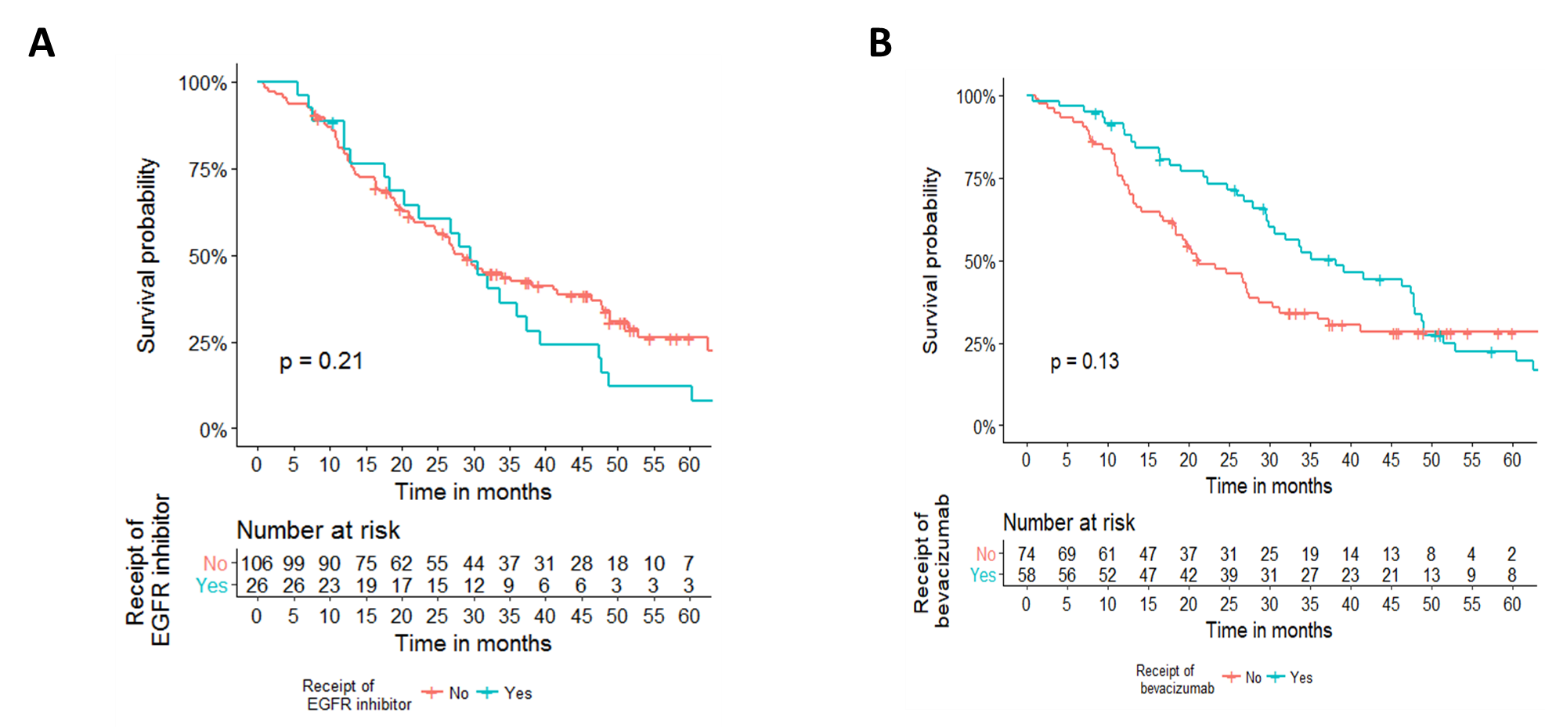
**

**A. Impact of receipt of EGFR inhibitor on OS among right-sided tumors.** Five-year OS 26% versus 12% no receipt versus receipt, respectively, p=NS.

**B. Impact of receipt of bevacizumab on OS among right-sided tumors.** Five-year OS 28% versus 22% no receipt versus receipt, respectively, p=NS.

**Supplemental Figure 5S.**

**
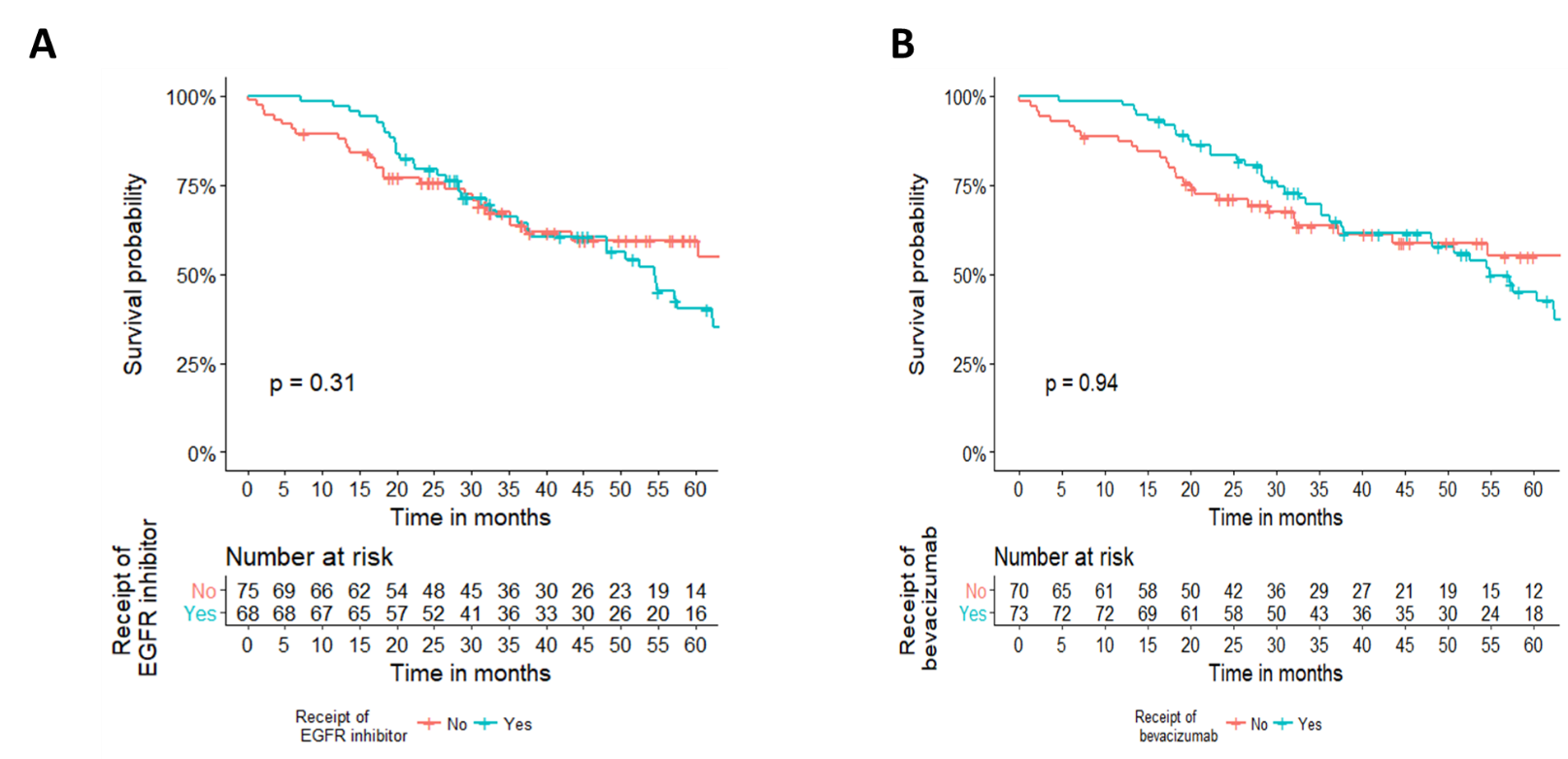
**

**A. Impact of receipt of EGFR inhibitor on OS among left-sided, *RAS* WT tumors.** Five-year OS 59% versus 40% no receipt versus receipt, respectively, p=NS.

**B. Impact of receipt of bevacizumab on OS among left-sided, *RAS* WT tumors.** Five-year OS 55% versus 45% no receipt versus receipt, respectively, p=NS.

**Supplemental Figure 6S.**

**
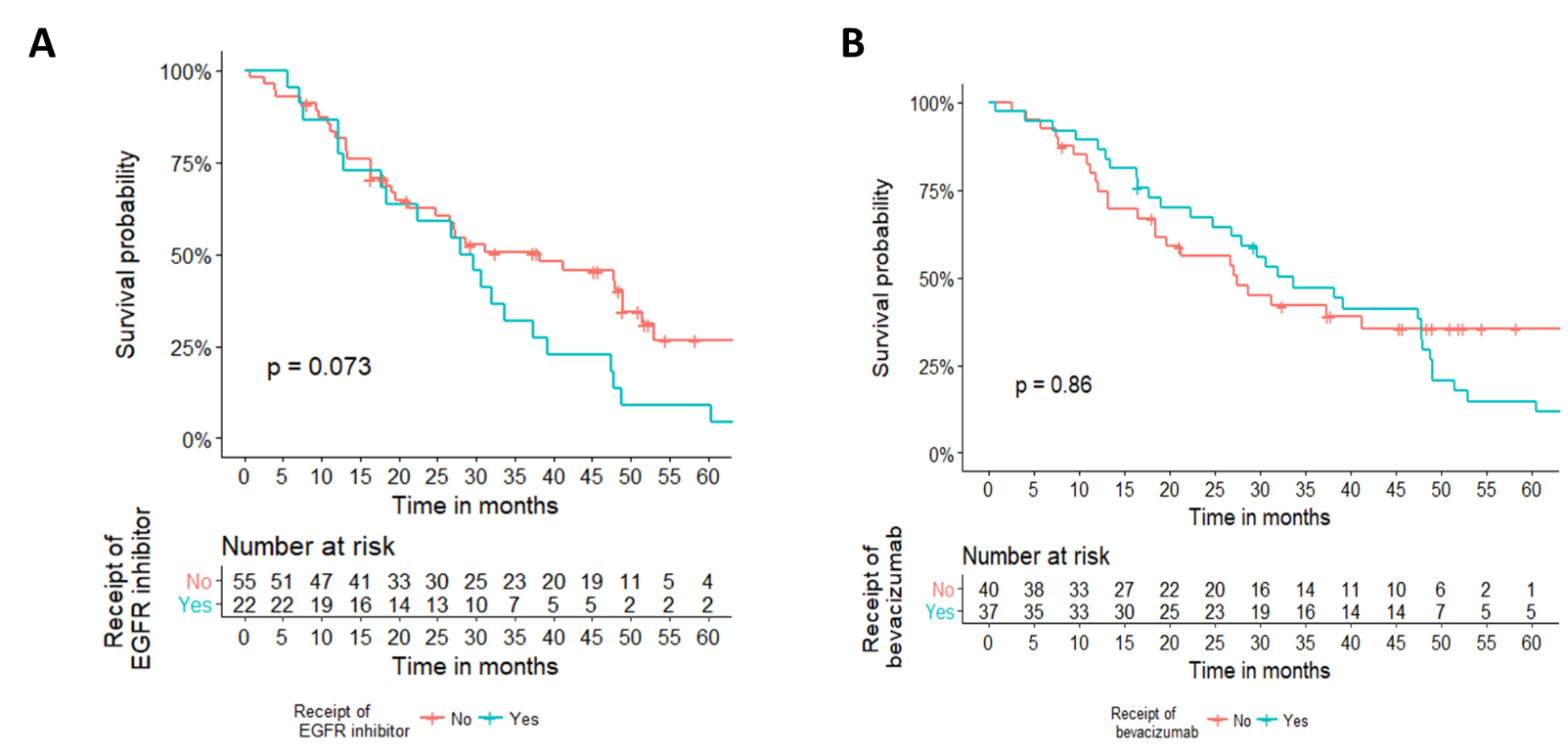
**

**A. Impact of receipt of EGFR inhibitor on OS among right-sided, *RAS* WT tumors.** Five-year OS 27% versus 9% no receipt versus receipt, respectively, p=NS.

**B. Impact of receipt of bevacizumab on OS among right-sided, *RAS* WT tumors.** Five-year OS 36% versus 15% no receipt versus receipt, respectively, p=NS.

**Supplemental Figure 7S.**

**
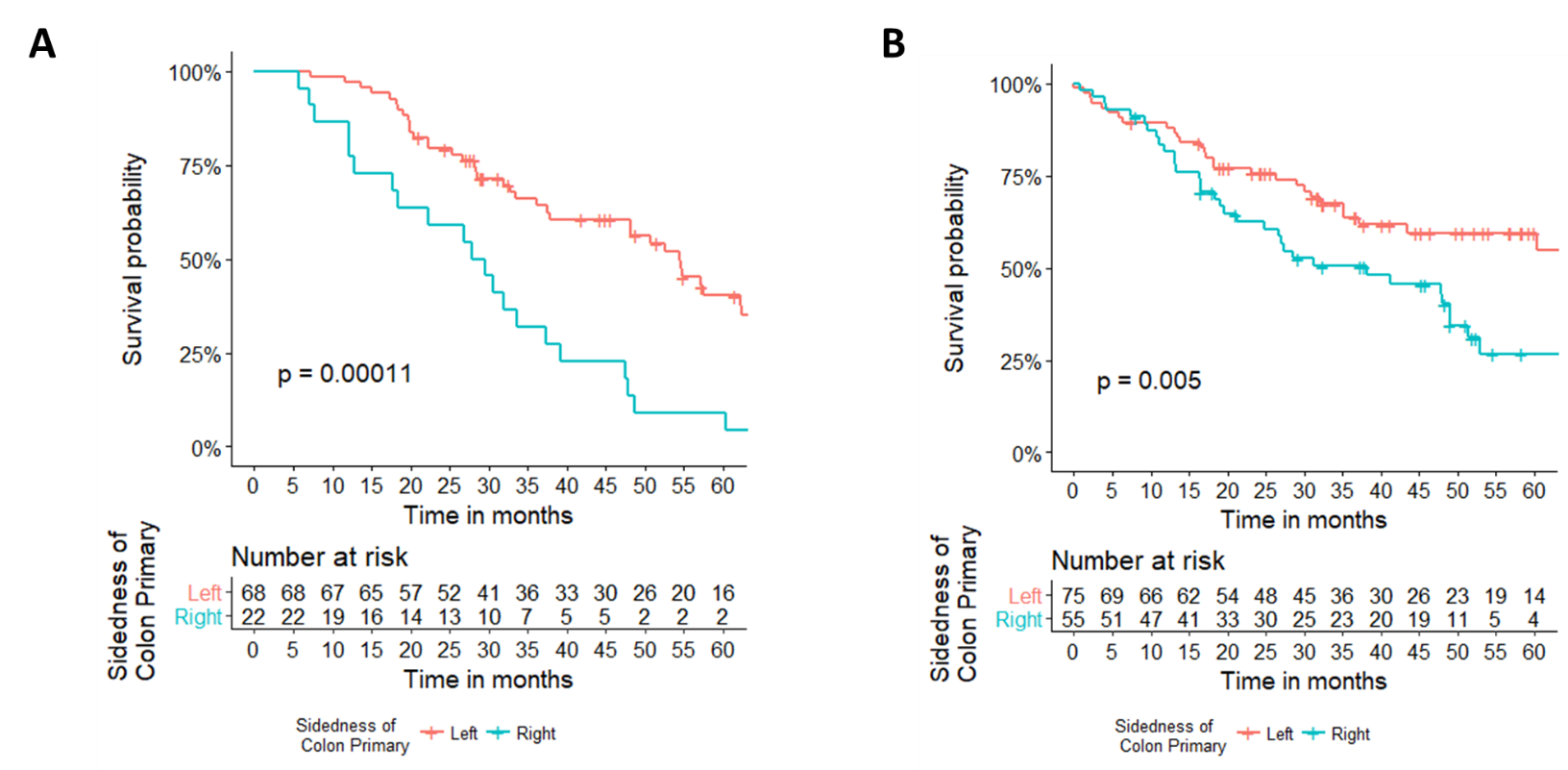
**

**A. Impact of EGFR inhibitor on OS by side among *RAS* WT patients.** Ninety *RAS* WT patients received EGFR inhibitor. Five-year OS 40% versus 9% left versus right respectively.

**B. Impact of no EGFR inhibitor on OS by side among *RAS* WT patients.** One hundred thirty *RAS* WT patients did not receive an EGFR inhibitor. Five-year OS 59% versus 27% left versus right respectively.

**Supplemental Figure 8S.**

**
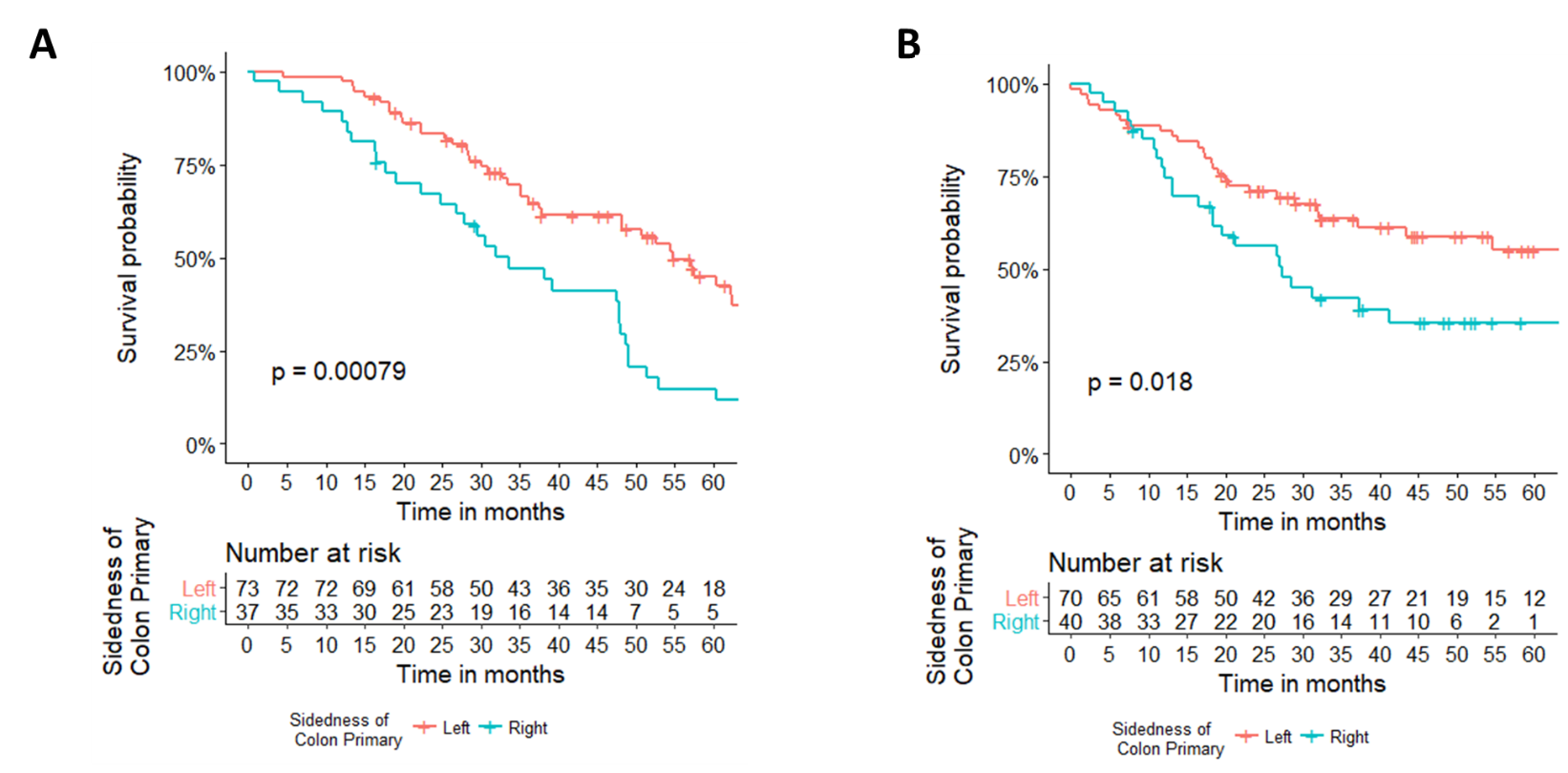
**

**A. Impact of bevacizumab on OS by side among *RAS* WT patients.** OS among those *RAS* WT patients who received bevacizumab. Five-year OS 45% versus 15%, left-sided versus right-sided tumors.

**B. Impact of no bevacizumab on OS by side among *RAS* WT patients.** OS among those *RAS* WT patients who did not receive bevacizumab. Five-year OS 55% versus 36% left-sided versus right-sided tumors.

**Supplemental Figure 9S.**

**
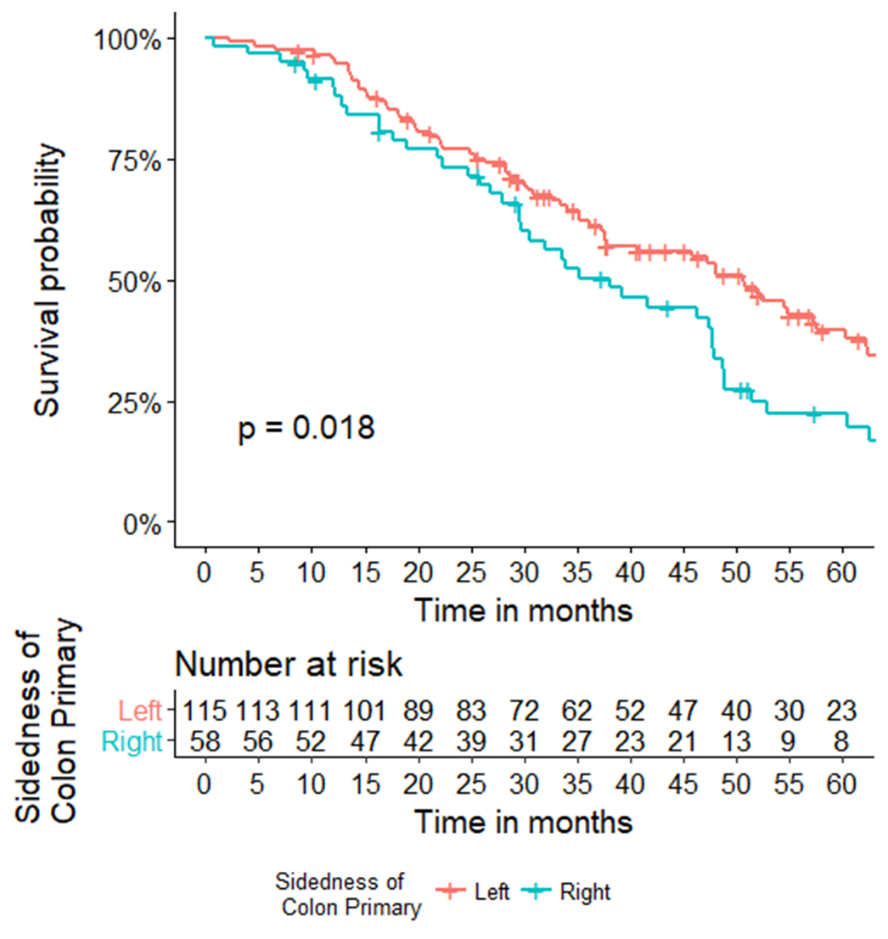
**

**Impact of bevacizumab on OS by side, regardless of *RAS* status.** Left-sided tumors had improved OS (HR 0.65, 95%CI 0.44-0.93, p=0.02) compared to right-sided tumors.
